# Prevention of Ophthalmia Neonatorum Caused by *Neisseria gonorrhoeae* Using a Fatty Acid-Based Formulation

**DOI:** 10.1128/mBio.00534-17

**Published:** 2017-07-25

**Authors:** Colin P. Churchward, Raid G. Alany, Ruth S. Kirk, Anthony J. Walker, Lori A. S. Snyder

**Affiliations:** School of Life Sciences, Pharmacy, and Chemistry, Kingston University, London, United Kingdom; Lahey Hospital and Medical Center

**Keywords:** antibiotic resistance, eye infection, fatty acids, gonococcus

## Abstract

Ophthalmia neonatorum, also called neonatal conjunctivitis, acquired during delivery can occur in the first 28 days of life. Commonly caused by the bacterial pathogen *Neisseria gonorrhoeae*, infection can lead to corneal scarring, perforation of the eye, and blindness. One approach that can be taken to prevent the disease is the use of an ophthalmic prophylaxis, which kills the bacteria on the surface of the eye shortly after birth. Current prophylaxes are based on antibiotic ointments. However, *N. gonorrhoeae* is resistant to many antibiotics and alternative treatments must be developed before the condition becomes untreatable. This study focused on developing a fatty acid-based prophylaxis. For this, 37 fatty acids or fatty acid derivatives were screened *in vitro* for fast antigonococcal activity. Seven candidates were identified as bactericidal at 1 mM. These seven were subjected to irritation testing using three separate methods: the bovine corneal opacity and permeability (BCOP) test; the hen’s egg test—chorioallantoic membrane (HET-CAM); and the red blood cell (RBC) lysis assay. The candidates were also tested in artificial tear fluid to determine whether they were effective in this environment. Four of the candidates remained effective. Among these, two lead candidates, monocaprin and myristoleic acid, displayed the best potential as active compounds in the development of a fatty acid-based prophylaxis for prevention of ophthalmia neonatorum.

## IMPORTANCE

Ophthalmia neonatorum is a painful bacterial infection of the eye in newborns. A common cause is *Neisseria gonorrhoeae*, which has the potential to cause severe damage to the eye and blindness. This study was designed to identify new and unconventional drug candidates that could be used in an eye ointment to prevent the development of this disease. The drugs that were tested were fatty acid-based compounds. The potential candidates were screened for their ability to kill bacteria in tear fluid without causing damage to the eye. This study identified seven candidates that are fast acting and nonirritating, with two strong candidates for potential use in the eye. Further development and testing are now required to formulate an eye treatment incorporating these drugs to prevent ophthalmia neonatorum.

## INTRODUCTION

"Ophthalmia neonatorum" is the term used to describe conjunctivitis occurring during the first 28 days of life; the condition is also referred to as neonatal conjunctivitis (1). The disease is characterized by inflammation of the eye with swelling of the eyelids and a purulent yellowish discharge from the closed eye. The infection may start in one eye and then progress to the contralateral one. If left untreated, or if standard treatment fails, the infection can cause corneal scarring or corneal perforation which can result in permanent blindness (2). The disease is caused by transfer of infectious agents from the mother to the eyes of the neonate during passage though the birth canal (3), with the two most common etiological agents being *Neisseria gonorrhoeae* and *Chlamydia trachomatis*. Gonococcal ophthalmia neonatorum commonly has a faster onset than *C. trachomatis* ophthalmia neonatorum and is usually more aggressive (4). The expectant mother may not know that she harbors *N. gonorrhoeae*. Between 2001 and 2006, more than 50% of the women that attended selected genitourinary medicine clinics in England and Wales had no symptoms of *N. gonorrhoeae* infection (5).

The disease can be controlled in three ways: selective treatment of expectant mothers via prenatal screening; blanket prenatal antimicrobial treatment of all expectant mothers; and postnatal application of prophylaxis to all neonates within the first few hours after birth.

Postnatal prophylaxis can be applied in the form of an eye drop or eye ointment. Current prophylaxes include 1% tetracycline and 0.5% erythromycin ophthalmic ointments. However, the use of tetracycline-based ophthalmic ointments is in decline due to concerns over resistance. The prophylaxis previously used was 1% to 2% aqueous silver nitrate; however, its use was discontinued due to chemically induced neonatal conjunctivitis and ocular toxicity (6). The United States Preventive Services Task Force has classified the use of an ocular prophylaxis for prevention of ophthalmia neonatorum as “grade A,” which means that there is high certainty that the net benefit is substantial (7). They recommend that all newborns are given a prophylaxis within 24 h of birth. However, in the United Kingdom there has been no prophylaxis since the 1950s and, currently, prenatal screening via assessment of risk factors is used (8).

Since 2008, gonorrhea diagnoses in England have increased year after year and doubled between 2008 and 2013 (9). There were 29,291 new gonorrhea diagnoses in England in 2013. Moreover, antimicrobial resistance is a major concern with *N. gonorrhoeae*, with isolates emerging that are multidrug resistant (10). Third-generation cephalosporins are the recommended antimicrobials, in combination with azithromycin; however, the first resistant isolates were reported in 2011 (11). Since then, resistant isolates have been reported in North America and Europe (12, 13). In 2009, levels of resistance to penicillin, tetracyclines, and ciprofloxacin in England and Wales were 22%, 68%, and 35%, respectively (14).

The antimicrobial properties of certain fatty acids and fatty acid derivatives have been known for some time (15–17). Research on the antimicrobial effects of fatty acid on *N. gonorrhoeae* has also been published (18). A novel ocular prophylaxis for the prevention of ophthalmia neonatorum based on a fatty acid or fatty acid derivative would offer an alternative to current prophylaxes in the face of increasing resistance concerns. Blindness was a signiﬁcant concern with gonococcal ophthalmia neonatorum before the introduction of antibiotics, with 3% of affected babies suffering permanent blindness (8). In 1880, before the advent of antibiotics, up to 79% of children in institutions for the blind had suffered from gonococcal ophthalmia neonatorum (18). We have to prepare ourselves now with treatment options to serve as alternatives to common antimicrobials.

## RESULTS

### Screening of the organic acids.

To find potential candidates, 37 organic acids, esters, and salts were tested in a two-tiered screening process. These candidates were chosen based on the carbon chain lengths of the fatty acids, the presence or absence of saturation, and modifications to represent a diverse selection of organic acid candidates. The first screen aimed to determine the ability of each compound to inhibit the growth of *N. gonorrhoeae* strain NCCP11945 at 1 mM in GC solid media (Table 1). This strain was chosen due to its relatively recent isolation, its antibiotic resistance profile, and the availability of a complete genome sequence. Those candidates able to inhibit the growth of *N. gonorrhoeae* strain NCCP11945 were investigated in a second assay. The second assay tested the ability of each compound to cause at least a 4-log reduction in viable bacteria at 1 mM with a 2-min exposure time (Table 1). Those candidates able to reduce the number of viable bacteria in this short time period are promising antigonococcal agents. This process identified the following seven candidates that had strong bactericidal properties: lauric acid; tridecanoic acid; myristoleic acid; palmitoleic acid; linolenic acid; monocaprin; and sodium dodecanoate.

**TABLE 1:** Investigation of antigonococcal properties of organic acids

Chemical class	Organic acid^a^	Growth inhibition^b^	Log reduction at 1 mM^c^	MBC (mM)^d^
Saturated fatty acids	Caprylic/octanoic acid (8:0)	No		
	Capric acid/decanoic acid (10:0)	No		
	Undecanoic acid (11:0)	**Yes**	No; 0.2	
	**Lauric acid** (**12:0**)	**Yes**	**Yes**; **>6**	**0.75**
	**Tridecanoic acid (13:0)**	**Yes**	**Yes**; **>6**	**0.75**
	Myristic acid (14:0)	**Yes**	No; 1.5	
	Pentadecylic acid (15:0)	**Yes**	No; 0.5	
	Palmitic acid (16:0)	No		
	Heptadecylic acid (17:0)	No		
	Stearic acid (18:0)	No		
Monounsaturated fatty acids	**Myristoleic acid (14:1); *c*Δ9**	**Yes**	**Yes; >6**	**0.5**
	**Palmitoleic acid (16:1); *c*Δ9**	**Yes**	**Yes; >6**	**0.5**
	Oleic acid (18:1); *c*Δ9	**Yes**	No; 1.0	
	Elaidic acid (18:1); *t*Δ9	**Yes**	No; 0.5	
	Petroselinic acid (18:1); *c*Δ6	No		
	*trans*-Vaccenic acid (18:1); *t*Δ11	No		
	*cis*-Vaccenic acid (18:1); *c*Δ11	No		
	Erucic acid (22:1); *c*Δ13	**Yes**	No; 0.0	
Polyunsaturated fatty acids	Sorbic acid (6:2); *t*Δ2,4	No		
	Linoleic acid (18:2); *c*Δ9,12	**Yes**	No; 1.6	
	**Linolenic acid (18:3); *c*Δ9,12,15**	**Yes**	**Yes**; **>6**	**0.5**
	Arachidonic acid (20:4) *c*Δ5,8,11,14	**Yes**	NT	
Monoglycerides	1-octanoyl-rac-glycerol (C8MG)	**Yes**	No; 0.0	
	**Monocaprin (C10MG)**	**Yes**	**Yes**; **>6**	**0.5**
	Monolaurin (C12MG)	**Yes**	No; 3.4	
	Monomyristin (C14MG)	**Yes**	No; 0.6	
Dicarboxylic acids	Succinic acid	No		
	Adipic acid	No		
	Pimelic acid	No		
	Suberic acid	No		
	Azelaic acid	No		
	Sebacic acid	No		
Ricinoleic acid	Ricinoleic acid	**Yes**	No; 3.5	
Esters	Isopropyl myristate	No		
	Isopropyl palmitate	No		
Fatty acid sodium salt	**Sodium dodecanoate**	**Yes**	**Yes**; **>6**	**0.75**
	Sodium myristate	**Yes**	No; 0.5	

a The common name for each organic acid is listed, as well as the commonly used numerical identification of fatty acids based on carbon chain lengths. Bold highlights the top candidates.

b *N. gonorrhoeae* strain NCCP11945 did or did not grow on GC agar media containing a 1 mM concentration of the candidate.

c Data represent the calculated log reduction in the level of viable bacteria after exposure to a 1 mM concentration of the candidate for 2 min. NT, not tested.

d Minimum bactericidal concentration (MBC) data were estimated as the lowest concentration to give a mean log reduction value above 4 from a 2-min exposure time.

### Measurement of bactericidal action of the fatty acids.

**FIG 1:**
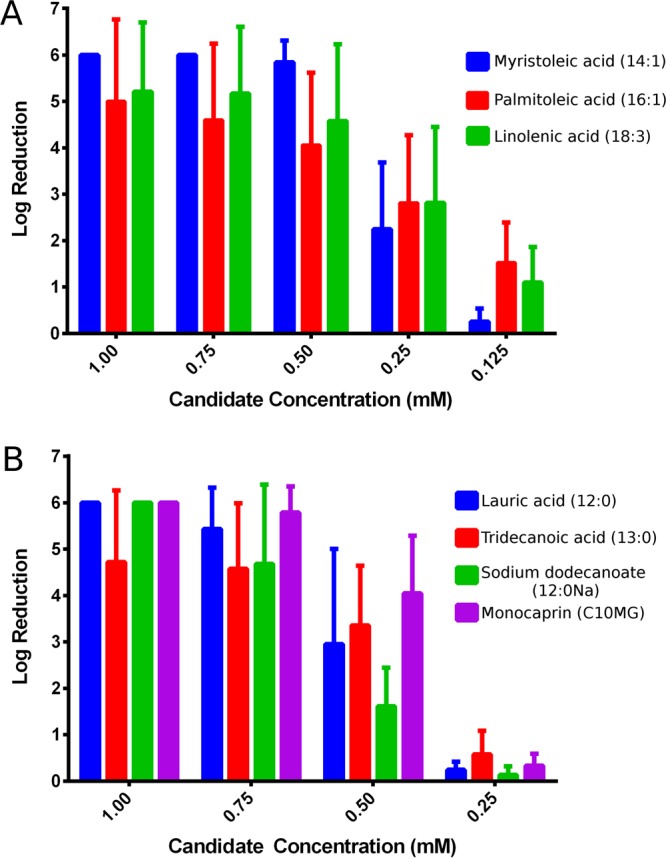
Log reduction assays conducted in culture medium with (A) unsaturated carbon chain fatty acids, and (B) saturated carbon chain fatty acids and fatty acid derivatives. Mean log reductions (± standard deviations [SD]) are shown for each of the seven candidates assayed against nine different gonococcal isolates. The maximum value for the log reduction was 6, so compounds with this value effectively killed all bacteria in the sample. The common name for each organic acid is used, as well as the commonly used numerical identification of fatty acids based on carbon chain lengths.

The seven candidates were further tested against nine gonococcal isolates, including strain NCCP11945 and eight isolates from Public Health England, to find their minimal bactericidal concentration (MBC). This was defined as the lowest concentration tested to give an average 4-log_10_ reduction in bacterial count after a 2-min exposure. Myristoleic acid, palmitoleic acid, linolenic acid, and monocaprin performed well with an MBC of 0.5 mM (Table 1). Lauric acid, tridecanoic acid, and sodium dodecanoate had an MBC of 0.75 mM. Figure 1 displays the log reduction graphs for each of the seven candidates tested against the nine isolates investigated. Any candidate that killed all gonococcal cells in the cell suspension was given a log reduction score of six. Lauric acid, myristoleic acid, monocaprin, and sodium dodecanoate tested at 1 mM killed all gonococcal cells for all isolates. However, palmitoleic acid, linolenic acid, and tridecanoic acid did not. Two gonococcal isolates in particular displayed some tolerance of palmitoleic acid and linolenic acid and were not completely inhibited by these compounds at 1 mM.

### Log reductions of selected fatty acids in artificial tear fluid.

**FIG 2:**
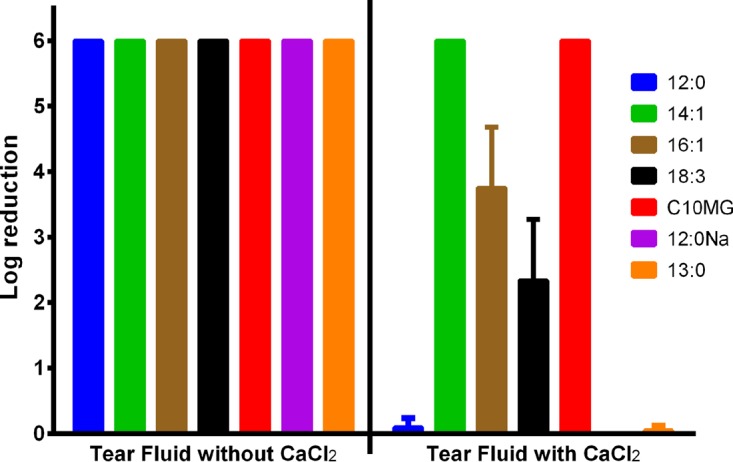
Log reductions in tear fluid without or with calcium (1 mM). Calcium present in the artificial tear fluid inactivated the antimicrobial effects of lauric acid (12:0), sodium dodecanoate (12:0Na), and tridecanoic acid (13:0). The calcium ions also affected palmitoleic acid (16:1) and linolenic acid (18:3) but to a lesser extent. Myristoleic acid (14:1) and monocaprin (C10MG) were unaffected by the presence of calcium in the artificial tear fluid.

The antigonococcal properties of the seven selected fatty acids were tested in artificial tear fluid, which contained calcium chloride. The addition of 1 mM lauric acid, tridecanoic acid, or sodium dodecanoate gave no reduction in bacterial cell number after exposure, in contrast to the results seen when they were tested in culture medium (Fig. 2). The tear fluid was also made without calcium chloride, and the antigonococcal properties of these fatty acids were completely restored (Fig. 2), indicating that calcium was having an inhibitory effect upon the candidates. This was most likely due to the negative effect of calcium on the aqueous solubility of these candidates.

### Ocular irritation assays.

**FIG 3:**
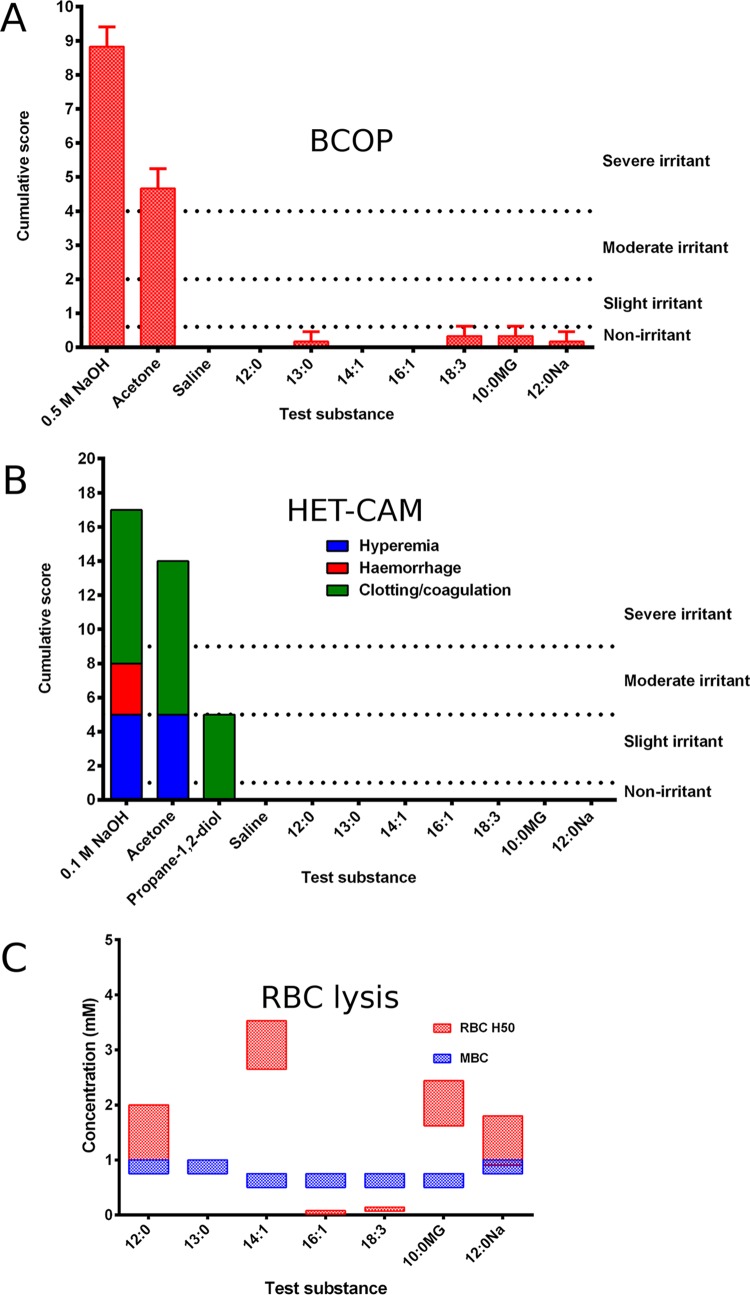
Summarized results of the irritation assays. (A) BCOP assay; 0.5 M NaOH and acetone were irritant controls, whereas saline solution was a nonirritant control. All candidates were nonirritants based on cumulative scores. (B) HET-CAM assay; 0.1 M NaOH, acetone, and propane-1,2-diol were irritant controls, and saline solution was a nonirritant control. In all cases, hyperemia, hemorrhage, and clotting/coagulation were scored. All candidates scored zero. (C) Combined results of assays of the RBC lysis, the concentration to lyse 50% of RBCs (H_50_), and the minimum bactericidal concentration (MBC) from Table 1. Tridecanoic acid did not lyse RBCs at any concentration investigated. Two candidates had MBC concentrations greater than their H_50_ results. Abbreviations: lauric acid (12:0); tridecanoic acid (13:0); myristoleic acid (14:1); palmitoleic acid (16:1); linolenic acid (18:3); monocaprin (10:0MG); and sodium dodecanoate (12:0Na).

**FIG 4:**
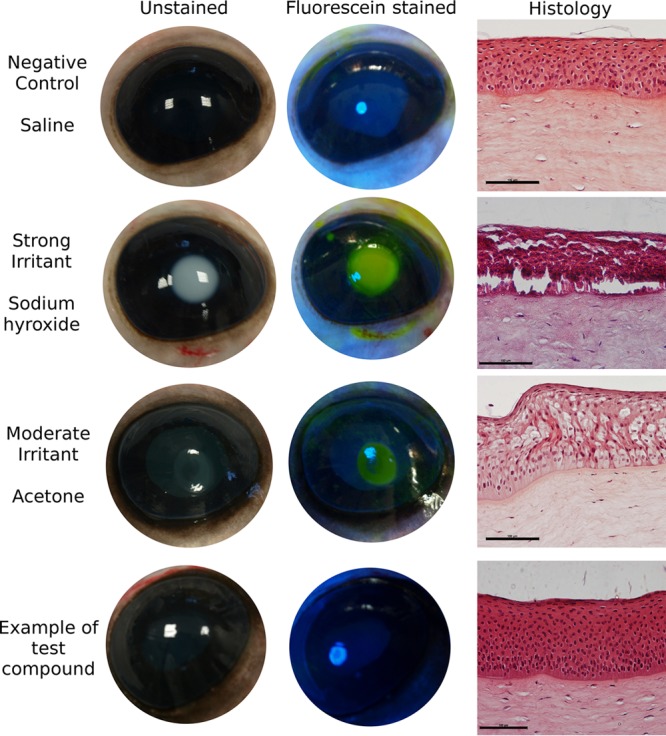
Unstained and fluorescein-stained bovine eyes exposed to components used in the BCOP test. Positive controls for irritation (sodium hydroxide and acetone) indicate the opacity of the cornea, and staining reveals changes in permeability. Histology micrographs (right) show hematoxylin and eosin (H&E)-stained sections of the epithelial layer of the cornea. Positive controls show extensive cytopathic damage to the epithelial cell layer, with complete detachment in the case of sodium hydroxide. The negative control with nonirritating saline solution and all of the candidate organic acids showed no opacity of the cornea, no notable staining with fluorescein, and no damage to the tissues revealed by histology. Images for sodium dodecanoate are shown as an example for all candidates. The scale shown on the histology images is equivalent to 100 µm.

The summary of the irritation assays is given in Fig. 3. The hen’s egg test—chorioallantoic membrane (HET-CAM) assay is an acceptable conjunctival model, and the bovine corneal opacity and permeability (BCOP) assay addresses corneal irritation potential (19–21). The seven candidates did not cause significant damage to the surface of the cornea as judged by fluorescein staining and histological examination (Fig. 4). Each of the organic compounds was tested on three separate occasions, and only linolenic acid, monocaprin, and sodium dodecanoate gave any sign of fluorescein staining. This staining occurred around the area where the rubber application ring was placed. However, the mean cumulative BCOP scores of these compounds were lower than 0.5, classing them as nonirritants.

**FIG 5:**
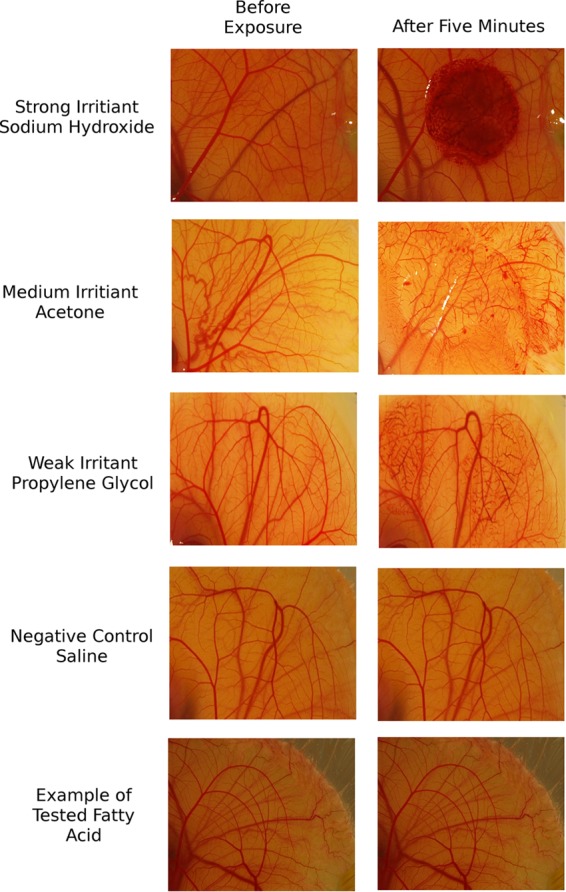
Photographs from HET-CAM test. Eggs were grown for 9 days, and then the tested components were spotted onto the chorioallantoic membrane for 5 min. The top six images show positive controls where the irritants caused hyperemia and clotting at the site of application upon comparison before application (left) and 5 min after application (right). These changes were not seen in the negative control when saline solution was applied or in the tested candidates, where application did not cause a reaction. Application of lauric acid (12:0) is shown in the bottom pair of images as an example for all candidates.

The samples were all tested on the HET-CAM at 1 mg/ml, providing a range of 3.6 mM for linolenic acid to 5 mM for lauric acid. As all candidates were bactericidal at 1 mM, these concentrations were used to demonstrate that these candidates would be safe to use. Results from the controls and an example of a tested candidate are illustrated in Fig. 5.

The red blood cell (RBC) assay is a more general irritation assay, investigating lysis of red blood cells as an assay for lipid bilayer disruption. The longer unsaturated fatty acids, palmitoleic acid and linolenic acid, required the smallest amount to cause 50% hemolysis (H_50_), and their H_50_ concentrations were lower than their minimum bactericidal concentration (MBC) (Fig. 3). Tridecanoic acid did not cause any hemolysis even at the highest concentration tested. The 12-carbon unsaturated sodium salt sodium dodecanoate gave results similar to those seen with lauric acid. Tridecanoic acid, myristoleic acid, monocaprin, and lauric acid all had H_50_ concentrations greater than their MBCs.

### Cell culture model of infection.

**FIG 6:**
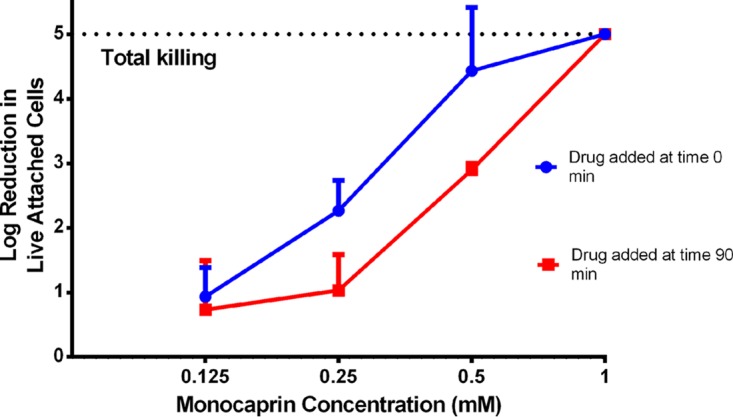
Log reduction of levels of *Neisseria gonorrhoeae* attached to human cornea epithelial cells when monocaprin was added immediately after the bacteria or 90 min after infection. Monocaprin delivered at 1 mM killed the bacteria even when added 90 min after inoculation. Mean log reductions are shown (±SD; *n* = 3).

Because of its potency, resistance to precipitation in the presence of calcium, and nonirritant nature, monocaprin was chosen from the seven candidates to test in a cell culture model with a 2-fold dilution series from 1 mM to 0.125 mM. Bacteria were also challenged with this compound at two different time points, i.e., at the time of the inoculation and 90 min later. Monocaprin killed all *N. gonorrhoeae* bacteria in coculture with human corneal epithelial cells even after the 90 min of infection (Fig. 6). There was a slight difference in the level of efficacy when monocaprin was used at sub-MBC levels and added 90 min after bacterial inoculation in comparison to addition immediately after inoculation of the bacteria (Fig. 6). This was possibly due to monocaprin hindering attachment of the bacterial cells to the corneal cells when it was added at the time of infection.

The mean log reduction results obtained in cell culture (Fig. 6) were similar to those obtained in bacterial cell suspensions (Fig. 2). This suggests that the action of monocaprin is concentration dependent, rather than time dependent, as the action of the drug during a 3-h exposure appeared similar to that seen in the original 2-min log reduction experiments.

When the drug was employed at 1 mM, there was no difference in the mean log reduction between addition of the drug at the time of infection and addition 90 min later, both demonstrating complete killing of *N. gonorrhoeae*. At 0.125 mM, however, the drug was completely ineffective at both time points. Bacterial log reductions showed that the concentrations of 0.25 and 0.5 mM were more effective at 0 min.

**FIG 7:**
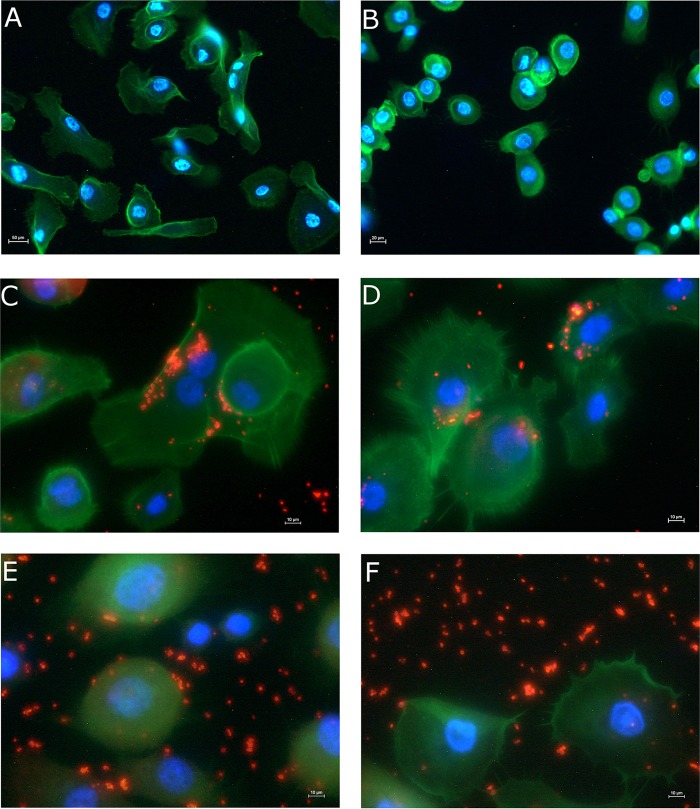
Immunofluorescent staining of human corneal epithelial cells with and without *Neisseria gonorrhoeae* infection. (A to D) Controls used were noninfected (A); noninfected and treated (with 1 mM monocaprin) (B); the infection control (C); and gentamicin treated (D). (E and F) The results presented in panels A to D were compared to results from wells treated with the drug candidate monocaprin at 1 mM (E) and 0.5 mM (F). Cellular actin was stained with phalloidin-Fluor 488 (green); *Neisseria gonorrhoeae* cells were stained with antigonococcal primary antibody and Alexa Fluor 555 secondary antibody (red); nuclei are stained with DAPI (blue). Images are representative of results observed in two experiments.

Cells exposed to the drug candidate and the controls were also assessed via fluorescence microscopy (Fig. 7). Comparing the images of the noninfected control and noninfected treated control, it appears that the monocaprin had a minor effect on the morphology of the cells. Corneal cells treated with 1 mM or 0.5 mM monocaprin appeared to be less spread and more spherical; this was not observed at lower drug concentrations (data not shown).

**FIG 8:**
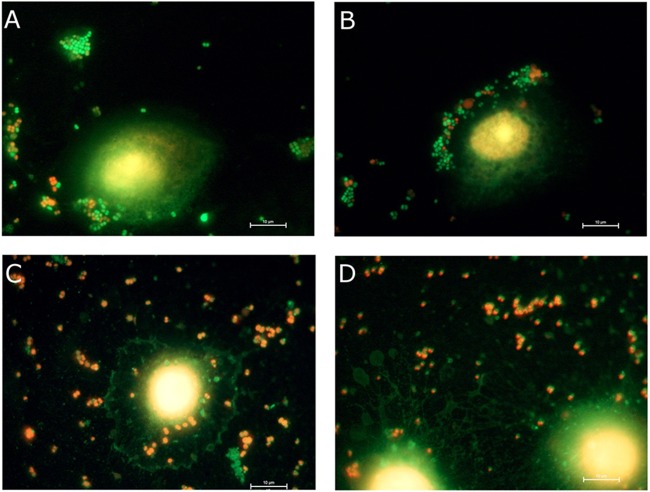
LIVE/DEAD staining of gonococcus-infected human corneal epithelial cells. Live bacteria appear green, while dead cells appear red. (A and B) Untreated infection control samples. (C and D) Human corneal epithelial cells infected and treated with 1 mM monocaprin. The untreated infection control sample had many green-staining live bacteria and a few dead bacteria stained red (A and B), whereas the monocaprin-treated samples contained only red-stained dead bacterial cells, some of which were still attached to the human corneal epithelial cells (C and D). Some green structures also appear in the treated samples, but these are likely to represent cell debris that was void of nuclear material stained by the red propidium iodide, giving the red appearance to dead bacterial cells. No viable bacteria were recovered from these wells. Images are representative of results observed in two experiments.

LIVE/DEAD staining of the corneal cells with and without infection revealed that the infection control samples contained a mixture of live and dead *N. gonorrhoeae* bacteria (Fig. 8), as would be expected from an inoculum generated from an overnight culture. In contrast, infected corneal cells that had been treated with monocaprin and subjected to LIVE/DEAD staining showed only dead bacterial cells (Fig. 8). These results give a clearer indication of the impact of the presence of monocaprin on the survival of the bacteria in a cellular model system than the results obtained with fluorescence microscopy alone.

## DISCUSSION

Among thirty-seven potential candidates included in the initial screen, seven candidate organic acids were found to have potent antimicrobial properties against *N. gonorrhoeae*. These were lauric acid, tridecanoic acid, myristoleic acid, palmitoleic acid, linolenic acid, monocaprin, and sodium dodecanoate. These seven fatty acids were tested in three irritation assays, using two gold standards for ocular drug therapy development, i.e., the BCOP assay and HET-CAM (19–21), and a more general RBC lysis assay (22), which may be less informative for development of ocular therapies. All seven candidates were shown to be effective in two of these assays (the BCOP assay and HET-CAM; Fig. 2), while five candidates were shown to be effective in the third test as well (the RBC lysis assay; Fig. 2). The MBC values for palmitoleic acid and linolenic acid were greater than their H_50_ values. However, it is unclear whether the values of the RBC lysis assay and the MBC are directly comparable. The RBC lysis assay is done by incubating the RBC with the candidate for 1 h, whereas the log reductions, used to estimate the MBC, employ a 2-min exposure regimen. Palmitoleic acid and linolenic acid also had not performed as well as the other candidates in the log reductions against all of the *N. gonorrhoeae* isolates.

Log reduction assays conducted in artificial tear fluid revealed that the saturated fatty acids lauric acid, tridecanoic acid, and sodium dodecanoate were ineffective in the presence of calcium ions. The inhibition of the antimicrobial effects of fatty acids by calcium ions was reported previously but could be limited by lowering the pH (23, 24). However, it is not recommended to have a low pH in an ocular formulation and the saturated fatty acids were therefore excluded from further analysis. After testing was performed in artificial tear fluid and irritation assays, the two best candidates highlighted for further development were monocaprin and myristoleic acid.

Use of a cell culture to model *N. gonorrhoeae* infection is well established (25). The cell culture work in this study demonstrates that monocaprin at 1 mM killed the gonococcal cells even when added after 90 min. For any drug candidate to be effective in a prophylaxis, it must clear the infectious agent from the surface of the eye. Most prophylaxes for ophthalmia neonatorum are applied in the first hour after birth. To be sure that a candidate could be used in a prophylaxis, the candidate must be able to kill the bacterial cells after the bacteria have had sufficient time to infect cells of the human eye. Importantly, in the current research, monocaprin was capable of killing *N. gonorrhoeae* bacteria even after they had infected the corneal cells for 90 min. Monocaprin had been reported previously to have strong antigonococcal properties (18), but myristoleic acid had not been tested. However, myristoleic acid was found to be ineffective against *C. trachomatis*, while monocaprin has again been shown to be bactericidal (26). Thus, monocaprin would have the advantage of being active against other causes of ophthalmia neonatorum. Monocaprin has also reportedly been used to make hydrogel-based formulations and has been proposed for the treatment of cold sores and as protection against sexually transmitted infections (27–29). Monocaprin-based hydrogels have been used in a mouse model for protection against herpes simplex virus 2 infection (29), and no irritation of the mouse vaginal mucosa was observed upon application of the hydrogel. Monolaurin has been shown to be a powerful anti-*Staphylococcus aureus* agent and has been tested as an additive to tampons in trials to control *S. aureus* growth to prevent exotoxin production (30). Antimicrobial resistance did not develop even after continuous passage on sublethal concentrations of monolaurin for a year (31).

The mechanism of action of monocaprin and myristoleic acid is unknown, but it has been suggested to involve cell membrane disruption (16–18). This would explain why these candidates caused lysis of RBCs. However, some candidates had MBCs far below their H_50_ results. This mechanism of action would have the added advantage that it is unlikely that the bacteria would develop resistance. Added to this is the way in which the infection develops, with neonatal ocular infections occurring after inoculation of the eyes during birth. Not only does prophylaxis to prevent ophthalmia neonatorum occur before biofilm formation, but also dispersion of biofilms by fatty acids has been shown previously (32). *N. gonorrhoeae* does possess at least two efflux systems (the *mtrCDE* and *farAB* systems) that can transport hydrophobic molecules out of the cell (33, 34). However, despite *N. gonorrhoeae* strain NCCP11945 possessing these efflux systems, it was unable to transport these fatty acids outside the cell quickly enough to prevent membrane lysis.

Fluorescence microscopy revealed that the higher concentrations of monocaprin influenced the morphology of the corneal cells. Morphology changes due to exposure to high levels of monocaprin have previously been reported (16, 35). However, one study that looked at the effect of 1.3 mM monocaprin exposure on the permeability of a Caco-2 monolayer demonstrated that the cells returned to normal after 30 h (35). *N. gonorrhoeae* cells remain visible in fluorescence microscopy after treatment; however, LIVE/DEAD staining clearly demonstrated that all of the gonococci had stained red, indicating that they were killed by the treatment with monocaprin (Fig. 8).

To fully establish whether monocaprin or myristoleic acid or both can be used in an ocular formulation to prevent ophthalmia neonatorum, the safety of these compounds has to be evaluated. Three irritation assays have been competed in this study, but an *in vivo* test will have to be conducted to be sure that no irritation occurs. There is no doubt that these two candidates have powerful antimicrobial properties that are stable in tear fluid, but their effectiveness on the surface of the eye needs to be fully evaluated.

## MATERIALS AND METHODS

### Bacterial strains and culture conditions.

The well-characterized *N. gonorrhoeae* NCCP11945 strain (36) was used in the screening stage. Eight isolates obtained from Public Health England, representing circulating isolates from various European locations, were used in the log reduction assays. All isolates were cultured for 24 h prior to testing by plating on GC solid medium (Oxoid Limited, Hampshire, United Kingdom) with Kellogg supplements and incubation at 37°C and 5% CO_2_ (37).

### Chemicals.

A list of all the organic acids tested in this study is presented in Table 1. The tested compounds include saturated and unsaturated fatty acids, monoglycerides, dicarboxylic acids, and fatty acid sodium salts. All compounds investigated were resuspended at 100 mM in ethanol unless stated otherwise. All chemicals were obtained from Sigma-Aldrich (Poole, Dorset, United Kingdom).

### Measurement of bactericidal action of selected fatty acids.

Log reductions were done as described by Bergsson and coworkers (18). Briefly, cells were suspended in GC broth (18 g/liter GC base [Oxoid, Limited] with added defined supplements) to an optical density at 520 nm of 0.3, which is approximately equivalent to 10^7^ to 10^8^ cells per ml (38). The suspension (500 µl) was then added to 5 µl of each 100 mM fatty acid stock to give a final assay concentration of 1 mM or to 5 µl of ethanol (vehicle control). The contents of the tubes were then mixed at room temperature for 2 min and immediately diluted in a 10-fold dilution series to 10^−4^. The dilutions were plated by spreading 10 µl on a quarter of a plate; a 100-µl neat solution was also plated. Plates were then incubated at 37°C for 48 h, with all samples plated in duplicate. A reduction in viable cell count by more than 4 log_10_ was deemed bactericidal, and the compound responsible was selected for further investigation. Bactericidal compounds were then tested at 0.75, 0.5, and 0.25 mM, with 0.125 mM also used for some potent compounds, to establish the minimum bactericidal concentration. Additional testing at lower concentrations was also done on the eight clinical isolates of European origin.

### Bovine corneal opacity and permeability (BCOP) test.

Bovine eyes were collected from a local slaughter house (ABP, Guildford, Surrey, United Kingdom) and were transported in cold saline solution for testing at the laboratory. Each eye was first visually examined for signs of damage before use. The test was conducted as detailed by Abdelkader and coworkers (21). The exposure time of the test substances was 30 s. The selected bactericidal compounds were tested at 1 mM in saline solution, with a corresponding vehicle control (1% ethanol–saline solution) also performed. A medium irritant of 100% acetone and a strong irritant of 0.5 M sodium hydroxide were also used as controls in each experiment. The test area of the cornea was then washed with 10 ml saline solution. Corneal damage to the test area was assessed by fluorescein staining and examination under a cobalt blue filter. Pathological scoring based on opacity, epithelial integrity, and epithelial detachment was done in accordance with previous publications (19). Each of the seven lead candidates was tested in triplicate, and each test was performed on a separate day using a different batch of eyeballs. Corneal histology was done as outlined by Abdelkader and coworkers (21) by evaluating the damage to the epithelial layer and stroma of the cornea after hematoxylin and eosin staining.

### Hen’s egg test—chorioallantoic membrane (HET-CAM).

The HET-CAM was performed as described by Alany and coworkers (20). Fertilized white leghorn eggs (Henry Stewart and Co. Ltd., Fakenham, Norfolk, United Kingdom) were checked for damage and incubated at 37°C with 60% to 70% relative humidity. At day 4, the eggs were removed from their shells and placed in incubation chambers. At day 9, the CAMs were tested. This was done by applying 200 µl of the testing material and monitoring inflammatory responses at 30, 120, and 300 s. At each of these time points, the conjunctival irritation potential of the test substance on the CAMs was evaluated by assessing them against the published scoring system (20).

### Red blood cell (RBC) lysis assay.

Whole bovine blood was obtained from a local slaughter house (ABP), adding 90 ml blood to 10 ml 110 mM trisodium citrate anticoagulant. RBCs were isolated the same day. The RBCs were washed by three repeats of centrifugation (1,500 × *g* for 10 min) and resuspension in phosphate-buffered saline (PBS) with 10 mM glucose (PBSG). Testing was done following the guidelines of the European Union Reference Laboratory for alternatives to animal testing (EURL-ECVAM) (22). The organic acids were solubilized in dimethyl sulfoxide (DMSO) and diluted to a maximum DMSO concentration of 10%. In a 2-ml centrifuge tube, 750 µl of the sample was added to 250 µl RBCs. Controls used in the assay were a complete lysis control (deionized water) and a fragility control (PBSG–10% DMSO). Samples were shaken at room temperature for 1 h and then centrifuged at 14,000 × *g* for 2 min. An aliquot of 750 µl of the supernatant was taken and its absorbance at 451 nm measured.

### Testing in artificial tear fluid.

Artificial tear fluid was made as described by Mirejovsky and others (39) with or without calcium chloride. The fatty acid candidates were tested in artificial tear fluid to demonstrate that they could kill the bacteria in this environment. As with the previous log reductions, 10^7^ gonococcal cells (isolate NCCP 11945) were suspended in GC broth. The cell suspension was centrifuged to remove the culture medium, and the bacteria were resuspended in the tear fluid. This was then added to the concentrated organic acid candidates. The cells were then enumerated again after 2 min of exposure to estimate any reduction in cell number due to the exposure to the organic acid.

### Cell culture infection model.

Primary cornea epithelial cells (Life Technologies, Inc.) were grown in keratinocyte serum-free medium (Life Technologies, Inc.) at 37°C with 5% CO_2_ until 80% confluence was reached. Cells were treated with trypsin, washed, added to 24-well plates, and incubated for a further 24 h. The medium was replaced and approximately 10^7^ CFU/ml P^+^ and Opa^+^ *N. gonorrhoeae* strain NCCP11945 added to reach a host-to-pathogen ratio (multiplicity of infection) of 1:10. Monocaprin (1 mM, 0.5 mM, 0.25 mM, or 0.125 mM) was added either at 0 min or at 90 min after seeding with *N. gonorrhoeae*. Controls included noninfected, noninfected treated (with 1 mM monocaprin), infection, and gentamicin controls. After 3 h, the cells were washed, treated with 1% saponin, and incubated for 10 min at 37°C. GC broth was added to each well, and log reductions were calculated as detailed above. Comparisons of the log reductions at different drug administration times at given concentrations were performed using an unpaired *t* test and GraphPad Prism (version 6.01).

For microscopy, cells were grown in 24-well plates containing 13-mm-diameter round glass coverslips and were treated as described above. After washing, cells were fixed in 4% paraformaldehyde for 20 min, washed in PBS, and blocked with 5% bovine serum albumin–PBS for 30 min. After washing was performed, rabbit polyclonal IgG antigonococcal antibody (Abcam, Inc.) (1/400 in PBS) was added and the reaction mixture was incubated at 4°C overnight. Wells were washed, goat polyclonal anti-rabbit-IgG conjugated to Alexa Fluor 555 (1/500) and phalloidin-Fluor 488 (Abcam, Inc.) (1/1,000) was added for 1 h, and then the reaction mixture was washed and mounted with Fluoroshield mounting medium with 4′,6-diamidino-2-phenylindole (DAPI) (Abcam, Inc.). Slides were viewed using a Nikon Eclipse i80 fluorescence microscope (Nikon United Kingdom) and images captured using standard DAPI, tetramethyl rhodamine isocyanate (TRITC), and TRITC filters and NIS-Elements BR 3.0 SP7 (Nikon United Kingdom) software. The images were later merged using ImageJ (40).

### LIVE/DEAD staining.

The LIVE/DEAD BacLight bacterial viability kit for microscopy (Life Technologies, Inc.) was used according to manufacturer’s instructions. After the course of infection, the culture wells were washed five times with sterile 0.85% NaCl to remove nonadhered bacterial cells. Stain was added to each well for 15 min at room temperature before washing in 0.85% saline solution was performed. Glass coverslips were mounted in BacLight mounting oil and visualized on a Nikon Eclipse i80 fluorescence microscope (Nikon United Kingdom) as detailed above.

### Ethics statement.

Fertilized white leghorn eggs for medical research were purchased from Henry Stewart and Co. Ltd. (Fakenham, Norfolk, United Kingdom). The eggs were used at day 4 and at day 9. In accordance with United Kingdom and EU ethics guidelines, experiments were terminated before day 10 of embryonic development and thus did not require licensing or approvals.
